# Exfoliation of Hexagonal Boron Nitride (h-BN) in Liquide Phase by Ion Intercalation

**DOI:** 10.3390/nano8090716

**Published:** 2018-09-12

**Authors:** Danae Gonzalez Ortiz, Celine Pochat-Bohatier, Julien Cambedouzou, Mikhael Bechelany, Philippe Miele

**Affiliations:** 1 Institut Européen des Membranes (IEM), UMR-5635, CNRS, ENCSM, University of Montpellier, Place Eugene Bataillon, 34095 Montpellier, France; danae.gonzales-ortiz@umontpellier.fr (D.G.O.); celine.pochat@umontpellier.fr (C.P.-B.); 2 Institut de Chimie Séparative de Marcoule (ICSM), CEA, CNRS, ENSCM, University of Montpellier, 30207 Marcoule, France; julien.cambedouzou@enscm.fr; 3 Institut Universitaire de France (IUF), 1 Rue Descartes, 75231 Paris CEDEX 5, France

**Keywords:** liquid exfoliation, boron nitride nanosheets, ion intercalation, gelatin

## Abstract

A green approach to prepare exfoliated hexagonal boron nitride nanosheets (h-BNNS) from commercially pristine h-BN involving a two-step procedure was investigated. The first step involves the dispersion of pristine h-BN within an aqueous solution containing gelatin and potassium or zinc chloride using a sonication method. The second involves the removal of larger exfoliated h-BNNS through a centrifugation procedure. The exfoliation was caused not only by the sonication effect but also by intercalation of K^+^ and Zn^2+^ ions. Transmission electronic microscopy, X-ray diffraction and Raman spectroscopy techniques show that the obtained h-BNNS generally display a thickness of about a few (2–3) layers with an exfoliation efficiency as high as 16.3 ± 0.4%.

## 1. Introduction

Two-dimensional (2D) nanosheets have been receiving great attention in recent years, due to their ultrathin structure with large planar dimensions and their outstanding properties [1,2,3]. The advantage of these materials is that they have great potential in a huge range of applications at scientific and technological levels due to their exfoliated state, as single- or few-layers. The most widely studied 2D layered material is graphene. It is ideally composed of a one-atom thick planar sheet of *sp*^2^ hybridized carbon atoms. Recently, other 2D layered materials, such as layered transition metal dichalcogenides (LTMDs) (e.g., MoS_2_ and WS_2_), metal oxides and hexagonal boron nitride (h-BN) have gained a renewed interest. Their lamellar structure can be subjected to exfoliation leading to single-layer and few-layers nanosheets. The structure and morphology of exfoliated 2D-layered materials strongly influence their properties; they present high surface area and quantum confinement effects [4,5] and also high chemical stability as well as insulating properties [6].

In this study, we focus our attention on h-BN. Its layered structure consists of a lattice similar to graphite in which boron (B) and nitrogen (N) atoms alternate in a hexagonal arrangement within the layer whereas they are in an eclipsed configuration when observed perpendicularly. Therefore, boron nitride nanosheets (BNNSs) are basically composed of sheets of *sp*^2^ hybridized 2D layers, organized in honeycomb geometry, with an interlayer distance of ca. 0.33–0.34 nm. Recently, the interest of scientists in BNNSs as a 2D nanomaterial has increased because of their advantages compared to graphene. Besides the unique chemical and thermal stability of BNNS [7,8], they also present great mechanical strength and electrical insulating properties (bandgap of 5~6 eV) [9]. Therefore, BNNSs have important applications in nanodevices or functional composites.

The aim of this study is to develop a new exfoliation method to obtain mono-layered or few-layered BNNS, minimizing the use of organic solvents and yielding a large amount of material. Nowadays, two different approaches exist for large-scale production of BNNS, bottom-up and top-down processes. The bottom-up approach includes chemical vapor deposition [10,11,12] and segregation methods [13]. This methodology provides single-layer BNNS but requires extreme conditions of temperature and pressure. Therefore, it is widely considered as unsuitable to synthesize BNNS on larger scales. On the other hand, the top-down approaches are based on exfoliating bulk h-BN crystals via mechanical [14,15,16] or sonication methods [17,18,19]. Such approaches have already been reported for graphene, for which individual sheets of 2D crystals were successfully separated. However, the B–N bond presents a partially ionic character compared to the covalent C–C bonding of graphene, leading to interactions between adjacent concentric shells or neighboring BN layers (so-called “lip-lip” interaction) [7].

Liquid-phase exfoliation is also one of the most investigated methods for exfoliating h-BN. Different attempts to prepare h-BNNS have been reported, such as treating bulk h-BN in various organic solvents. Zhi et al. dissolved h-BN in DMF applying vigorous ultrasonication followed by exfoliation. The exfoliation is attributed in this case to the strong interactions between DMF and the BN surface [18]. Warner et al. used 1,2-dicholoethane to exfoliate bulk h-BN by ultrasonic bath [19]. Zhou et al. demonstrated that they can obtain a highly stable suspension of exfoliated BN using a mixture of solvents such as water and ethanol [20]. Coleman et al. proposed a number of different solvents based on Hansen solubility to exfoliate h-BN. They found the best results with N-methylpyrrolidone (NMP) and isopropanol (IPA) [21]. Ye et al. exfoliated bulk h-BN powder in chloroform under sonication using hyperbranched polyethylene (HBPE) as stabilizer. They obtained stable dispersion of h-BNNS in chloroform [22]. Griffin et al. used an aqueous solution of sodium chlorate combined with ultrasonic power to exfoliate h-BN. The resultant solution was subjected to a centrifugation cascade to isolate smaller nanosheets [23]. Furthermore, molten hydroxides have been used to exfoliate h-BN. Sodium hydroxide (NaOH), potassium hydroxide (KOH) and BN were ground, then they were transferred into an autoclave and heated up to 180 °C. BNNS were obtained as a result [24]. Using these methods, a small yield of around 5% could be achieved. Besides organic solvents, water has been used as a solvent for exfoliation of h-BN using ultrasounds. Gelatin-assisted h-BN exfoliation under ultrasonic conditions has been also reported without large success concerning the exfoliation yield [25]. All these approaches failed to produce h-BNNS with high-yield exfoliation and in an environmentally friendly way. Therefore, a new low-cost method to produce large scale and high-quality h-BNNS needs to be explored.

Herein, we present a facile and green procedure combining sonication and centrifugation methods to produce high-yield h-BNNS from a commercially available powder. Gelatin and chloride salts were used to assist the exfoliation. The ion intercalation between the layered structures of bulk material facilitates the exfoliation. The obtained h-BNNS generally displays a thickness of about a few (3–9) layers with an exfoliation efficiency as high as 16.3%, and has high crystallinity.

## 2. Materials and Methods

### 2.1. Materials

Hexagonal boron nitride (h-BN) was supplied from Saint Gobain (Cavaillon, France) (CAS No. 10043-11-5, 95% purity, 325 mesh, 3 µm particle size). Zinc chloride (ZnCl_2_, 99.99% trace metal basis), potassium chloride (KCl, ≥99%) and gelatin type A (gel strength 300) were purchased from Sigma Aldrich (St. Quentin Fallavier, France). In all the experiments deionized pure water (18 MΩ) was used.

### 2.2. Exfoliation of h-BN

Hexagonal boron nitride (h-BN) nanosheets were fabricated using liquid-phase exfoliation with the assistance of an ultrasound device (BANDELIN, Berlin, Germany) (model SONOPLUS HD 3100, 100 W, 20 kHz) with a 3 mm diameter microtip (MS73). 1.0 g of pristine h-BN was added to 100 mL of water. The solution was heated up to 80 °C, then 20 g of gelatin were added. The mixture was kept under stirring at the same temperature until complete dissolution of the gelatin. Different concentrations of ZnCl_2_ and KCl were added to the mixture. The dispersion was kept in a bath at 50 °C to avoid the gelatin solidification and it was sonicated for 3 h at 65% amplitude with pulse off/on 0.5–1 s. After sonication, the yellowish suspension was subjected to two centrifugation steps. In a first step, the solution was centrifuged at 3000 rpm for 30 min. This first centrifugation step ensures the separation of large h-BNNS from light ones by remaining at the bottom of the solution. Then, the supernatant was collected and subjected to a second centrifugation step at 6000 rpm for 30 min. The supernatant was collected and dried at 60 °C overnight. In a first step, the resultant material was heated up to 600 °C under air to remove the gelatin. In a second step, the obtained powders were heated up to 1000 °C under Ar atmosphere with a flux of 200 mL/min, to improve the h-BNNS crystallinity. The obtained h-BNNS were washed several times with water and ethanol to remove any remaining impurities.

### 2.3. Characterization Techniques

The transmission electron microscopy (TEM) was performed using a JEOL 2200 FS-200 kV equipped with a STEM (Scanning Transmission Electron Microscopy, JEOL, Tokyo, Japon) module a Bright Field (BF) detector and a CCD Gatan USC 4092x4092px^2^ camera. To carry out the analysis, a drop of h-BNNS/isopropanol dispersion was deposited on the surface of the carbon grids. Atomic force microscopy (AFM) analysis was performed using an AFM NANOMAN 5 from Veeco Instrument controlled with the Nanoscope V software V8 (Bruker Corporation, Coventry, UK). The exfoliated h-BN was previously dissolved in water and then a drop of the solution was deposited on a silicon wafer. X-ray diffraction patterns of exfoliated h-BNNS were recorded using a PANalytical Xpert powder X-ray diffraction (XRD) system (PANalytical, Almelo, Hollande) with Cu Kα radiation, a scan speed of 2° min^−1^, a 2θ range between 3° and 70°, and a step rate about 0.02° per second. The Fourier transformed infrared (FTIR) spectra were recorded with a NEXUS instrument (Thermo Fisher Scientific, Waltham, USA) equipped with an attenuated total reflection (ATR) accessory in the frequency range of 600−4000 cm^−1^. Raman spectra have been obtained from a Horiba xplora, λ = 659 nm. The yield of exfoliated h-BNNSs was calculated from the following equation:

Yield (%) = (Weight (h-BNNS)/Weight (h-BN)) × 100(1)

## 3. Results

The starting h-BN powder has a typical lateral particle size in the range of approximately 3 µm. The preparation of h-BNNS was carried out from h-BN pristine in a simple combination of gelatin/chloride salts sonication and centrifugation steps. Additional heating steps were performed to remove the gelatin and improve the h-BNNS crystallinity. The yield of obtained h-BNNS was calculated from Equation (1). The results are shown in Table 1. The best exfoliation efficiency (16.3%) is obtained when 1.0 wt % of KCl was used to intercalate the ions within the layered structure of h-BN (Table 1).

### 3.1. Transmisson Electron Microscopy

**Figure 1:**
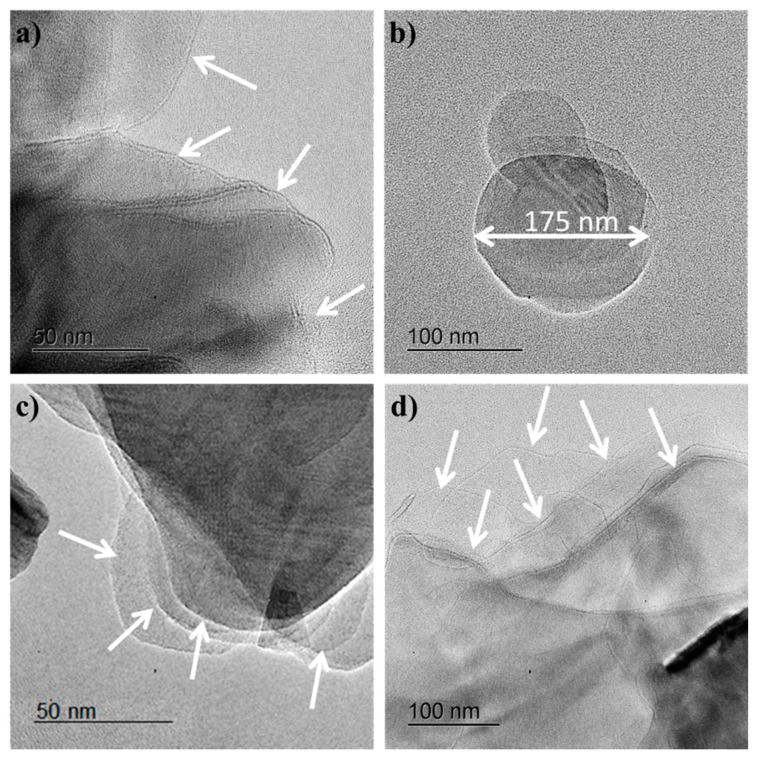
TEM images of h-BNNS exfoliated with (**a**) 0.5 wt % KCl; (**b**) 1.0 wt % KCl; (**c**) 0.5 wt % ZnCl_2_ and (**d**) 1.0 wt % ZnCl_2_.

Transmission electron microscopy (TEM) studies were carried out in order to determine the morphology of h-BNNS. The as-obtained dispersion showed the presence of irregularly shaped h-BNNS with lateral sizes ranging from a few tens of nanometers to as large as over 0.5 µm (Figure 1). The relative thickness of the h-BNNS could be visually examined by the transparency/darkness of the nanosheets against the electron beam, with thinner nanosheets appearing lighter. It is observed that the h-BNNS flakes obtained when the exfoliation is assisted by KCl (Figure 1a,b) display a lateral size mostly less than 200 nm, and the thickness is about few (2–3) layers. White arrows were added to the TEM images to facilitate the identification of the nanosheets. These results indicate that the sheets reduced their size by “cutting” the large h-BN sheets into smaller ones. Furthermore, the sonication process allows an effective “peeled out” of nanosheets from the bulk h-BN material, forming mono-layered and few-layered nanosheets with reduced lateral size in the dispersion, since the initial size of pristine h-BN is about 3 µm.

The nanosheets obtained when higher amounts of salts are used to exfoliate the pristine h-BN are thinner than when a lower concentration of salts is used. This could be due to the fact that at higher concentrations, more ions can be intercalated into the layered structure of h-BN, weakening the van der Waals interactions between the layers and leading to greater exfoliation.

In the case of h-BNNS obtained when the exfoliation is assisted by ZnCl_2_ (Figure 1c,d), the flakes display a lateral size up to 0.5 µm and are composed of a more significant amount of layers. The exfoliation efficiency using ZnCl_2_ is smaller compared to the exfoliation involving KCl. These results might be explained regarding ionic diameters. The ionic diameter of K^+^ is 2.76 Å while the ionic diameter for Zn^2+^ is 1.48 Å. Taking into account that the interlayer distance of pristine h-BN is 3.3 Å, both ions are able to intercalate between the h-BN layers, but K^+^ ions are larger than Zn^2+^ ions. Thus, the incorporation of K^+^ ions should have a higher ability to weaken the inter-sheet interactions and to destabilize the packing of BN sheets. This results in a higher exfoliation degree. This approach allows the reduction of the lateral size of h-BNNS as well as a reduction of the number of layers.

### 3.2. Atomic Force Microscopy

**Figure 2:**
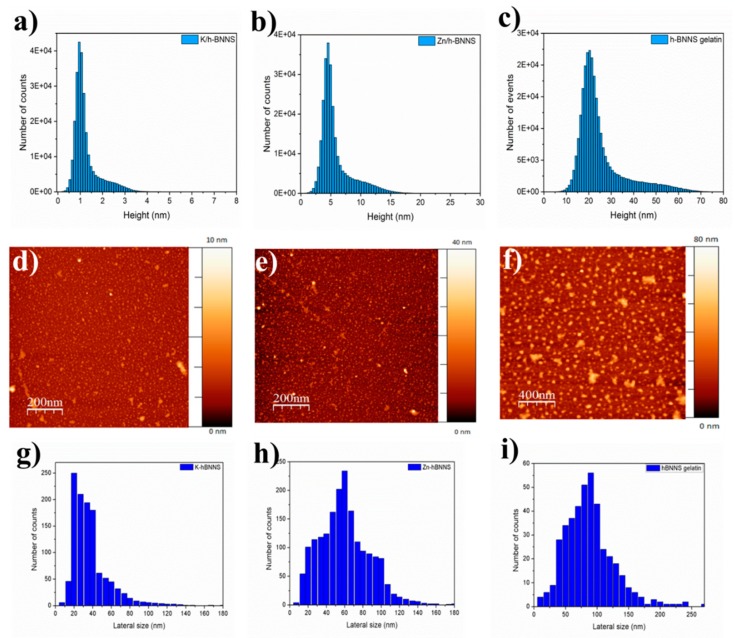
(**a**) Height of h-BNNS exfoliated with 1.0 wt % KCl; (**b**) Height of h-BNNS exfoliated with 1.0 wt % ZnCl_2_; (**c**) Height of h-BNNS exfoliated with gelatin; (**d**) AFM image of h-BNNS intercalated with 1.0 wt % KCl; (**e**) AFM image of h-BNNS intercalated with 1.0 wt % ZnCl_2_; (**f**) AFM image of h-BNNS intercalated with gelatin; (**g**) Lateral size of h-BNNS exfoliated with 1.0 wt % KCl; (**h**) of h-BNNS exfoliated with 1.0 wt % ZnCl_2_ and (**i**) Lateral size of h-BNNS exfoliated with gelatin.

h-BNNS obtained after liquid exfoliation in water assisted by gelatin and by gelatin with ZnCl_2_ or KCl using sonication tip, were characterized as well by AFM. A number of about 100 h-BNNS have been considered for each sample. The images (Figure 2) indicate a reduction on the thickness of pristine h-BN from 3 µm to 1–3 nm in the case of intercalation of K^+^ ions, and reduction of thickness to 2–6 nm for the intercalation of Zn^2+^ ions. Based on these AFM images and taking into account the width of one nanosheet (0.33 nm), we could assume that our h-BNNS are generally composed of 3–9 layers in the case of exfoliation with 1 wt % KCl. Besides, the h-BNNS exfoliated with 1 wt % ZnCl_2_ displays a thickness of about 6–48 nm, which corresponds to around 2–6 layers. If we compare these results with those when the exfoliation is assisted only with gelatin, we observe that the exfoliation degree is more important when ions assist the exfoliation than when only gelatin is added. The thickness, the number of layers and the lateral size of the nanosheets are summarized in Table 2.

Otherwise, when the exfoliation is assisted only with gelatin, we observed that the nanosheet size is reduced in comparison to pristine h-BN, from 3 µm to about 10–80 nm. If we compare these resulting nanosheets with the ones obtained when the exfoliation is assisted with gelatin/Zn^2+^ or gelatin/K^+^ ions, the exfoliation degree is much lower (Figure 2g–i) Furthermore, the h-BNNS obtained through ion intercalation are composed of around 3–9 layers of lateral size about 10–80 nm in case of exfoliation with 1 wt % KCl, while the h-BNNS resulting from gelatin exfoliation are composed of around 30–200 layers of lateral size 20–250 nm. Thus, we can say that the addition of ions to the solution during sonication helps to improve the exfoliation degree and to obtain nanosheets with smaller dimensions.

### 3.3. X-Ray Diffraction

**Figure 3:**
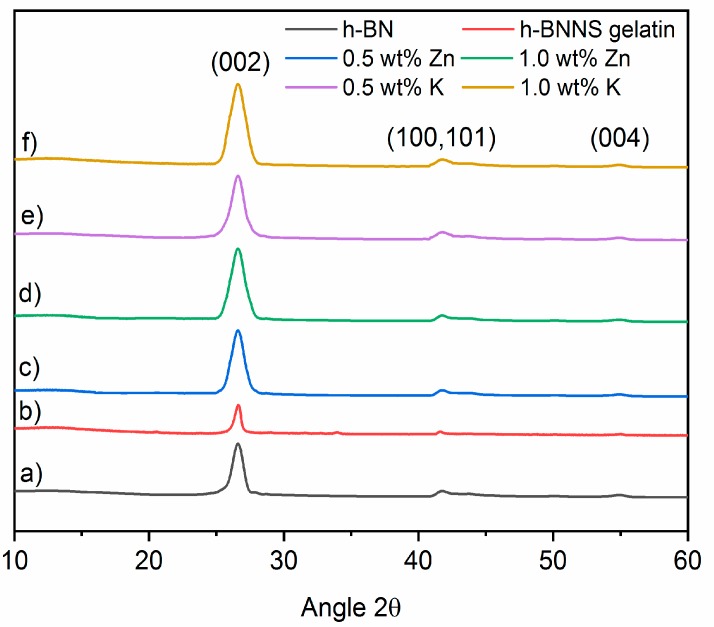
XRD patterns (**a**) pristine h-BN; (**b**) h-BNNS gelatin; (**c**) h-BNNS exfoliated by intercalation of 0.5 wt % ZnCl_2_; (**d**) h-BNNS exfoliated by intercalation of 1.0 wt % ZnCl_2_; (**e**) h-BNNS exfoliated by intercalation of 0.5 wt % KCl; and (**f**) h-BNNS exfoliated by intercalation of 1.0 wt % KCl.

X-ray diffraction (XRD) was used to investigate the phase structure of the as-prepared materials. h-BN (Figure 3) displays the diffraction peaks at 2θ = 26.66°, ~40–45° and 55.05° which can be correlated to the (002), unresolved (100) and (101), and (004) planes of the hexagonal phase of BN. The unresolved reflections will appear as (10×) in the graphics. No impurity from salts was detected by XRD after h-BNNS were washed (Figure S1), which indicates that the as-prepared h-BNNSs are pure. Energy-dispersive X-ray spectroscopy (EDX) analyses were performed to confirm the purity of obtained h-BNNS (Figure S2) (Tables S1 and S2). However, it is interesting to note that the 002 peak of h-BNNS, exfoliated via intercalation of Zn^2+^ or K^+^ ions, becomes broader regarding pristine h-BN.

The full width at half maximum (FWHM) of a peak is sensitive to the variation of the microstructure and stress-strain of the materials. For that, the FWHM of the 002 diffraction peak of h-BNNS was calculated from the XRD patterns. The results are shown in Table 3. It is observed that the FWHM increases when the h-BN is exfoliated with KCl and ZnCl_2_. The h-BNNS intercalated with 1 wt % of K^+^ and Zn^2+^ ions display a higher value of FWHM, indicating that the crystallite size might decrease during the exfoliation process. As is already known in the literature, the peak broadness is related to the crystallite size and it varies inversely with the FWHM: as the crystallite becomes smaller, the peak becomes broader [26]. For this reason, we also calculated the crystallite size of the h-BN pristine, the h-BN exfoliated with gelatin, and the h-BNNSs intercalated with Zn^2+^ and K^+^ at different concentrations using the Scherrer equation [27]. The results of the calculations are shown in Table 3.

The results obtained by the Scherrer equation show that increasing the salt (ZnCl_2_ and KCl) concentration during the intercalation results in smaller h-BNNS crystallites as compared to pristine h-BN. In the case of exfoliation assisted with gelatin only, the reduction of crystallite size is less pronounced. The initial crystallite size in pristine h-BN is calculated to be 20.6 nm. It has to be pointed out that the crystallite size calculated from Scherrer equation assumes that all crystallites have the same size and shape, and also that the crystallite size is different than the particle size (a particle may be made up of several different crystallites). This explains the differences emerging from the comparison of the results from XRD and AFM techniques. On one hand, the addition of the K^+^ ions during sonication shifts the peak to lower angles, from 2θ = 26.69° in pristine h-BN to 26.55° in h-BNNS. On the other hand, it reduces the crystallite size of the resulting h-BNNS down to 4.9 nm when 0.5 wt % KCl was used and down to 2.9 nm when 1.0 wt % KCl was introduced. In the case of intercalation with Zn^2+^ ions, the peak is also shifted to lower angles, from 2θ = 26.69° in pristine h-BN to 26.61 and 26.59° in h-BNNS intercalated with 0.5 and 1.0 wt % ZnCl_2_, respectively.

The crystallite size is also reduced in comparison to pristine h-BN: from 20.6 nm to 10.7 nm and 4.2 nm when 0.5 and 1.0 wt % ZnCl_2_ were introduced, respectively. In the case of h-BN exfoliation assisted by gelatin, the FWHM is slightly reduced if compared with pristine h-BN, thus crystallite size is also reduced from 20.6 nm to 20.1 nm. This shift of the peak position in the exfoliated h-BNNS is related to the interlayer distance (*d*-spacing). For this reason, the interlayer distance of h-BN and h-BNNS intercalated with 0.5 wt % and 1.0 wt % of KCl and ZnCl_2_ was calculated using Bragg law. The results are shown in Table 3. It is observed that the intercalation either with K^+^ or Zn^2+^ ions has an influence on the BN interlayer distance. Pristine h-BN displays a *d*-spacing of 0.334 nm. The intercalation of 0.5 wt % and 1.0 wt % of KCl gives a larger interlayer distance, i.e., 0.335 nm. Note that the increase of the interlayer distance is too small to be due to remaining ions between the layers. It is more probably due to structural defects coming from the sonication process in the BN planes that modifies their equilibrium distance.

On the other hand, the intercalation of Zn^2+^ ions produces the same effect; the interlayer distance is increased to 0.335 nm when 0.5 wt % and 1.0 wt % of ZnCl_2_ was used, respectively. In the case of the addition of gelatin alone, the interlayer distance also increases up to 0.335 nm but as we mentioned before, the crystallite size is larger than when the exfoliation is assisted with both gelatin and ions.

### 3.4. Raman Spectroscopy

**Figure 4:**
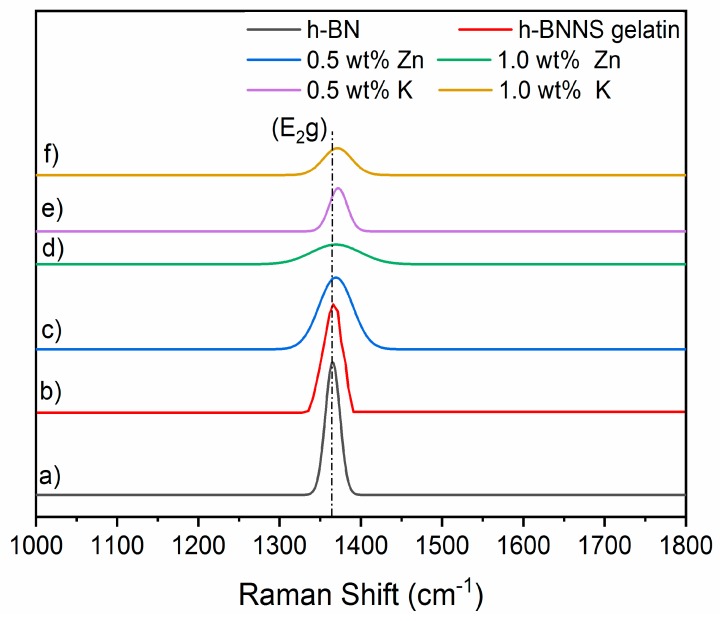
Comparative Raman spectra of (**a**) pristine h-BN; (**b**) h-BNNS gelatin; (**c**) h-BNNS exfoliated by intercalation of 0.5 wt % ZnCl_2_; (**d**) h-BNNS exfoliated by intercalation of 1.0 wt % ZnCl_2_; (**e**) h-BNNS exfoliated by intercalation of 0.5 wt % KCl; and (**f**) h-BNNS exfoliated by intercalation of 1.0 wt % KCl.

Raman spectra of pristine h-BN, h-BNNS gelatin and h-BNNS exfoliated with the intercalation of K^+^ ions are shown in Figure 4. h-BN and h-BNNS spectra exhibit a characteristic peak due to the typical B–N stretching mode (E_2g_). Table 4 shows the Raman shifts of pristine h-BN, h-BNNS gelatin and h-BNNS intercalated with 0.5 wt % and 1.0 wt % KCl and ZnCl_2_. Pristine h-BN displays this peak at ~1366 cm^−1^. It is observed that the peak intensity becomes progressively weaker and broader in both cases: exfoliation with gelatin and with ions intercalation. In addition, the increase of ion concentration makes this effect more pronounced. The exfoliation assisted with gelatin results in a small shift to higher wavenumbers of this peak at about 1367 cm^-1^. The exfoliation using 0.5 wt % and 1.0 wt % of KCl results in the shift of the E_2g_ phonon mode to approximately 1370 cm^−1^, corresponding to a blue shift of ~4 cm^−1^. In general, the shift in the peak position can be linked to strain conditions within the layers [11,28,29]. Thus, results agree with an effective exfoliation of h-BN into thinner flakes, which leads to a higher in-plane strain and weaker interlayer interactions [30,31,32]. This effect is more pronounced when increasing the ion concentration [33].

Raman spectra of h-BNNS exfoliated with the intercalation of Zn^2+^ ions are also shown in Figure 4. Similar to the exfoliation of h-BN with K^+^, the peak intensity becomes progressively weaker and broader as the ion concentration increases. The E_2g_ phonon mode of 0.5 wt % and 1.0 wt % of ZnCl_2_ is centered at approximately 1368 cm^−1^, corresponding to a ~2 cm^−1^ blue shift. In this case, the shift is lower than for the intercalation of K^+^. This fact is in line with the lower degree of exfoliation that has been evidenced by the others characterization techniques presented above.

### 3.5. Fourier-Transform Infrared Spectroscopy

**Figure 5:**
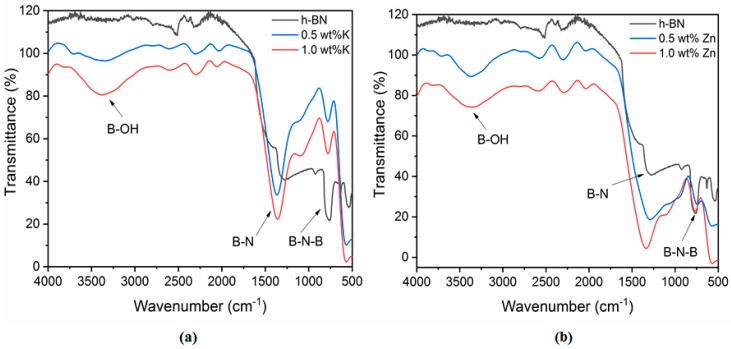
(**a**) FTIR spectra of pristine h-BN of and h-BNNS intercalated with Zn^2+^ at different concentrations (0.5 wt % and 1.0 wt % KCl); and (**b**) FTIR spectra of pristine h-BN of and h-BNNS intercalated with Zn^2+^ at different concentrations (0.5 wt % and 1.0 wt %).

Figure 5 shows the FTIR spectra of pristine h-BN and h-BNNS intercalated with K^+^ and Zn^2+^ ions. Two strong FTIR bands at ~1380 cm^−1^ and ~812 cm^−1^ are present in pristine h-BN, which are attributed to the B–N stretching (in-plane ring vibration, E_1u_ mode) and the B–N–B bending (out-of-plane vibration, A_2u_ mode), respectively. Table 5 shows the FTIR bands attribution and peak positions presented on the spectra. Intercalated h-BN with 0.5 wt % and 1.0 wt % KCl and ZnCl_2_ present the same bands than the pristine h-BN.

In addition, the FTIR spectra of h-BNNS intercalated with 0.5 wt % and 1.0 wt % KCl and ZnCl_2_ show another absorption peak at ~3200 cm^−1^, which could be ascribed to hydroxyl group (–OH) vibration. This peak might appear due to the large number of defects such as vacancy defects, dislocations and exposed edges introduced on the h-BNNS surfaces during sonication. Also, a new bending mode appears at ~1200 cm^−1^ which is correlated to (–OH) vibration.

The exfoliation of these materials can be explained by the effect of acoustic cavitation of high-frequency ultrasound in the formation, growth and collapse of microbubbles in solution. This effect induces shock waves on the surface of the bulk material causing exfoliation. In addition, the introduced ions (K^+^ and Zn^2+^) could be inserted into the h-BN layers, inducing an increase in the interlayer spacing. Furthermore, the non-polar chains of the gelatin can adsorb on the surface of flakes through hydrophobic–hydrophobic interactions, which results in the formation of a stable dispersion. Further research concerning reaction conditions, such as varying the reaction time, the applied power or the reaction temperature, might be performed to improve the reaction yield.

## 4. Conclusions

In summary, a green approach to prepare exfoliated h-BNNS from commercially pristine h-BN involving a two-steps procedure was investigated. The exfoliation was caused not only by sonication effect but was also assisted by gelatin and intercalation of K^+^ and Zn^2+^ ions. The latter resulted in the observation of few-layered h-BNNS of reduced lateral size, as well as few-layered h-BNNS with a lateral size below 200 nm, as evidenced from TEM images. The yield for h-BNNS intercalated with 1.0 wt % KCl was about 16.3 ± 0.4%. The crystallinity of the obtained h-BNNS was confirmed using XRD. Raman microscopy further confirmed the presence of the few-layered flakes, by analyzing the peak position shift from the pristine h-BN. The FTIR showed a band corresponding to –OH functions in the obtained h-BNNS through the intercalation of the ions. These functions could be attributed to the functionalization of h-BNNS edges or vacancy defects. Future works are in progress in order to improve the yield of the exfoliated h-BNNS by controlling the different factors that could affect the exfoliation, such as the size of pristine h-BN, the sonication conditions (time, power or pulse) or the centrifugation steps.

## Figures and Tables

**Table 1:** Yields of h-BNNS obtained through exfoliation with gelatin, and gelatin assisted ion intercalations with 0.5 and 1.0 wt % of KCl and ZnCl_2_.

Sample	Initial h-BN (g)	Purified h-BNNS (g)	Yield (%)
Gelatin	1.015	0.065	6.4 ± 0.1
0.5 wt % K/h-BNNS	1.014	0.143	14.1 ± 0.2
1.0 wt % K/h-BNNS	1.016	0.165	16.3 ± 0.4
0.5 wt % Zn/h-BNNS	1.005	0.124	12.3 ± 0.2
1.0 wt % Zn/h-BNNS	1.009	0.108	10.8 ± 0.2

**Table 2:** Summary of thickness of pristine h-BN and exfoliated h-BNNS.

Sample	Thickness	N° of Layers	Lateral Size (nm)
h-BN	3 µm	-	-
h-BNNS gelatin	10–70 nm	3–200	20–250
1.0 wt % Zn/h-BNNS	2–16 nm	6–48	10–120
1.0 wt % K/h-BNNS	1–3 nm	3–9	10–80

**Table 3:** Position of the 002 diffraction peak, *d* spacing and crystallite size of h-BN, h-BNNS-gelatin and h-BNNS intercalated with different concentrations of KCl and ZnCl_2._

Sample	Peak Position (°)	*d*-Spacing (nm)	FWHM β (°)	Crystallite Size (nm)
h-BN	26.69	0.334	0.4	20.6
h-BNNS gelatin	26.61	0.335	0.4	20.1
0.5 wt % K/h-BNNS	26.55	0.335	1.6	4.9
1.0 wt % K/h-BNNS	26.55	0.335	2.9	2.9
0.5 wt % Zn/h-BNNS	26.61	0.335	0.7	10.7
1.0 wt % Zn/h-BNNS	26.59	0.335	1.9	4.2

**Table 4:** Raman shift of h-BN, h-BN gelatin and h-BNNS intercalated with different concentrations of KCl and ZnCl_2_.

Sample	Raman Shift (cm^−1^)
h-BN	1365.7 ± 0.1
h-BNNS gelatin	1366.8 ± 0.3
0.5 wt % K/h-BNNS	1369.3 ± 0.4
1.0 wt % K/h-BNNS	1370.2 ± 0.2
0.5 wt % Zn/h-BNNS	1368.1 ± 0.2
1.0 wt % Zn/h-BNNS	1368.1 ± 0.3

**Table 5:** FTIR bands attribution and peak positions of h-BN and h-BNNS intercalated with different concentrations of KCl and ZnCl_2._

Sample	Attribution	Peak Position
h-BN	B–N stretching	1270.9
	B–N–B bending	759.7
0.5 wt % K/h-BNNS	O–H stretching	3372.9
	B–N stretching	1359.6
	B–N–B bending	779.7
1.0 wt % K/h-BNNS	O–H stretching	3340.2
	B–N stretching	1365.4
	B–N–B bending	779.1
0.5 wt % Zn/h-BNNS	O–H stretching	3355.6
	B–N stretching	1336.5
	B–N–B bending	767.5
1.0 wt % Zn/h-BNNS	O–H stretching	3367.2
	B–N stretching	1288.2
	B–N–B bending	742.5

## Supplementary Materials

The following are available online at http://www.mdpi.com/2079-4991/8/9/716/s1, Figure S1: XRD patterns of exfoliated h-BNNS in presence of ZnCl_2_ without further washing, Figure S2: (a) EDX image of h-BNNS exfoliated with 1.0 wt % ZnCl_2_; (b) Element mapping images of the h-BNNS exfoliated with 1.0 wt % ZnCl_2_, Table S1: EDX descriptive analysis of h-BNNS exfoliated with 0.5 wt % and 1.0 wt % KCl, Table S2: EDX descriptive analysis of h-BNNS exfoliated with 0.5 wt % and 1.0 wt % ZnCl_2_.

## References

[1] Novoselov K.S., Geim A.K., Morozov S.V., Jiang D., Zhang Y., Dubonos S.V., Grigorieva I.V., Firsov A.A. (2004). Electric field effect in atomically thin carbon films. Science.

[2] Huang X., Qi X., Boey F., Zhang H. (2012). Graphene-based composites. Chem. Soc. Rev..

[3] Huang X., Yin Z., Wu S., Qi X., He Q., Zhang Q., Yan Q., Boey F., Zhang H. (2011). Graphene-based materials: Synthesis, characterization, properties, and applications. Small.

[4] Mak K.F., Lee C., Hone J., Shan J., Heinz T.F. (2010). Atomically thin MoS_2_: A new direct-gap semiconductor. Phys. Rev. Lett..

[5] Nicolosi V., Chhowalla M., Kanatzidis M.G., Strano M.S., Coleman J.N. (2013). Liquid exfoliation of layered materials. Science.

[6] Xu M., Liang T., Shi M., Chen H. (2013). Graphene-like two-dimensional materials. Chem. Rev..

[7] Golberg D., Bando Y., Huang Y., Terao T., Mitome M., Tang C., Zhi C. (2010). Boron nitride nanotubes and nanosheets. ACS Nano.

[8] Chen Y., Zou J., Campbell S.J., Le Caer G. (2004). Boron nitride nanotubes: Pronounced resistance to oxidation. Appl. Phys. Lett..

[9] Kubota Y., Watanabe K., Tsuda O., Taniguchi T. (2007). Deep ultraviolet light-emitting hexagonal boron nitride synthesized at atmospheric pressure. Science.

[10] Shi Y., Hamsen C., Jia X., Kim K.K., Reina A., Hofmann M., Hsu A.L., Zhang K., Li H., Juang Z.-Y. (2010). Synthesis of few-layer hexagonal boron nitride thin film by chemical vapor deposition. Nano Lett..

[11] Song L., Ci L., Lu H., Sorokin P.B., Jin C., Ni J., Kvashnin A.G., Kvashnin D.G., Lou J., Yakobson B.I. (2010). Large scale growth and characterization of atomic hexagonal boron nitride layers. Nano Lett..

[12] Yu J., Qin L., Hao Y., Kuang S., Bai X., Chong Y.-M., Zhang W., Wang E. (2010). Vertically aligned boron nitride nanosheets: Chemical vapor synthesis, ultraviolet light emission, and superhydrophobicity. ACS Nano.

[13] Xu M., Fujita D., Chen H., Hanagata N. (2011). Formation of monolayer and few-layer hexagonal boron nitride nanosheets via surface segregation. Nanoscale.

[14] Pacile D., Meyer J., Girit Ç., Zettl A. (2008). The two-dimensional phase of boron nitride: Few-atomic-layer sheets and suspended membranes. Appl. Phys. Lett..

[15] Li L.H., Chen Y., Behan G., Zhang H., Petravic M., Glushenkov A.M. (2011). Large-scale mechanical peeling of boron nitride nanosheets by low-energy ball milling. J. Mater. Chem..

[16] Li L.H., Glushenkov A.M., Hait S.K., Hodgson P., Chen Y. (2014). High-efficient production of boron nitride nanosheets via an optimized ball milling process for lubrication in oil. Sci. Rep..

[17] Lin Y., Williams T.V., Connell J.W. (2009). Soluble, exfoliated hexagonal boron nitride nanosheets. J. Phys. Chem. Lett..

[18] Zhi C., Bando Y., Tang C., Kuwahara H., Golberg D. (2009). Large-scale fabrication of boron nitride nanosheets and their utilization in polymeric composites with improved thermal and mechanical properties. Adv. Mater..

[19] Warner J.H., Rummeli M.H., Bachmatiuk A., Büchner B. (2010). Atomic resolution imaging and topography of boron nitride sheets produced by chemical exfoliation. ACS Nano.

[20] Zhou K.G., Mao N.N., Wang H.X., Peng Y., Zhang H.L. (2011). A mixed-solvent strategy for efficient exfoliation of inorganic graphene analogues. Angew. Chem. Int. Edit..

[21] Coleman J.N., Lotya M., O’Neill A., Bergin S.D., King P.J., Khan U., Young K., Gaucher A., De S., Smith R.J. (2011). Two-dimensional nanosheets produced by liquid exfoliation of layered materials. Science.

[22] Ye H., Lu T., Xu C., Han B., Meng N., Xu L. (2018). Liquid-phase exfoliation of hexagonal boron nitride into boron nitride nanosheets in common organic solvents with hyperbranched polyethylene as stabilizer. Macromol. Chem. Phys..

[23] Griffin A., Harvey A., Cunningham B., Scullion D., Tian T., Shih C.-J., Gruening M., Donegan J.F., Santos E.J., Backes C. (2018). Spectroscopic size and thickness metrics for liquid-exfoliated h-BN. Chem. Mater..

[24] Pakdel A., Bando Y., Golberg D. (2014). Nano boron nitride flatland. Chem. Soc. Rev..

[25] Ge Y., Wang J., Shi Z., Yin J. (2012). Gelatin-assisted fabrication of water-dispersible graphene and its inorganic analogues. J. Mater. Chem..

[26] Cao L., Emami S., Lafdi K. (2014). Large-scale exfoliation of hexagonal boron nitride nanosheets in liquid phase. Mater. Express.

[27] Scherrer P. (1912). Bestimmung der Inneren Struktur und der Größe von Kolloidteilchen Mittels Röntgenstrahlen.

[28] Kim G., Jang A.-R., Jeong H.Y., Lee Z., Kang D.J., Shin H.S. (2013). Growth of high-crystalline, single-layer hexagonal boron nitride on recyclable platinum foil. Nano Lett..

[29] Gorbachev R.V., Riaz I., Nair R.R., Jalil R., Britnell L., Belle B.D., Hill E.W., Novoselov K.S., Watanabe K., Taniguchi T. (2011). Hunting for monolayer boron nitride: Optical and raman signatures. Small.

[30] Li L.H., Cervenka J., Watanabe K., Taniguchi T., Chen Y. (2014). Strong oxidation resistance of atomically thin boron nitride nanosheets. ACS Nano.

[31] Zhu W., Gao X., Li Q., Li H., Chao Y., Li M., Mahurin S.M., Li H., Zhu H., Dai S. (2016). Controlled gas exfoliation of boron nitride into few-layered nanosheets. Angew. Chem. Int. Edit..

[32] Cai Q., Scullion D., Falin A., Watanabe K., Taniguchi T., Chen Y., Santos E.J., Li L.H. (2017). Raman signature and phonon dispersion of atomically thin boron nitride. Nanoscale.

[33] Sainsbury T., Satti A., May P., Wang Z., McGovern I., Gun’ko Y.K., Coleman J. (2012). Oxygen radical functionalization of boron nitride nanosheets. J. Am. Chem. Soc..

