# Supplementary material for: Exfoliation of Hexagonal Boron Nitride (h-BN) in Liquide Phase by Ion Intercalation
Source: Nanomaterials (Basel). 2018 Sep 12;8(9):716. doi: 10.3390/nano8090716 (PMC6165242; doi:10.3390/nano8090716)
Supplement: Supplementary file 1 [file nanomaterials-08-00716-s001.pdf]

Supporting Information

Exfoliation of Hexagonal Boron Nitride (h-BN) in Liquide Phase by Ion Intercalation

Danae Gonzalez Ortiz [1], Celine Pochat-Bohatier [1], Julien Cambedouzou [2], Mikhael Bechelany [1,*] and Philippe Miele [1,3,*]

[1] Institut Européen des Membranes, IEM UMR-5635, ENCSM, CNRS, University of Montpellier, Place Eugene Bataillon, 34095, Montpellier, France; danae.gonzales-ortiz@umontpellier.fr (D.G.O.); celine.pochat@umontpellier.fr (C.P.-B.)

[2] ICSM, CEA, CNRS, ENSCM, University of Montpellier,30207, Marcoule, France; julien.cambedouzou@enscm.fr

[3] Institut Universitaire de France, IUF, 1 Rue Descartes, 75231, Paris, cedex 5, France

* Correspondences: Mikhael.bechelany@umontpellier.fr (M.B.); Philippe.miele@umontpellier.fr (P.M.); Tel.: +33-4-6714-9167 (M.B.)

Table S1. EDX descriptive analysis of h-BNNS exfoliated with 0.5 wt % and 1.0 wt % KCl.

Spectra descriptive	0.5 wt % KCl h-BNNS	1.0 wt % KCl h-BNNS
B	36	29
C	17	27
N	45	40
O	1.7	3
K	<1	<1
Total	100.00	100.00

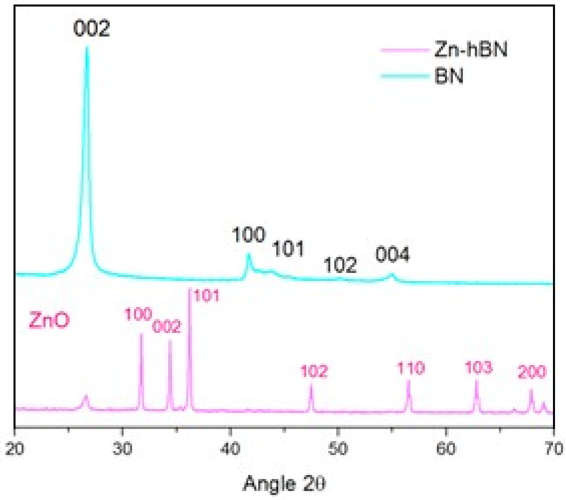

Figure S1. XRD patterns of exfoliated h-BNNS in presence of $ZnCl_2$ without further washing.

Table S2. EDX descriptive analysis of h-BNNS exfoliated with 0.5 wt % and 1.0 wt % $ZnCl_2$.

Spectra Descriptive	0.5 wt % $ZnCl_2$ h-BNNS	1.0 wt % $ZnCl_2$ h-BNNS
B	44	33
C	15	18
N	30	15
O	7	26
Na	<1	2
Zn	3	4
Total	100.00	100.00

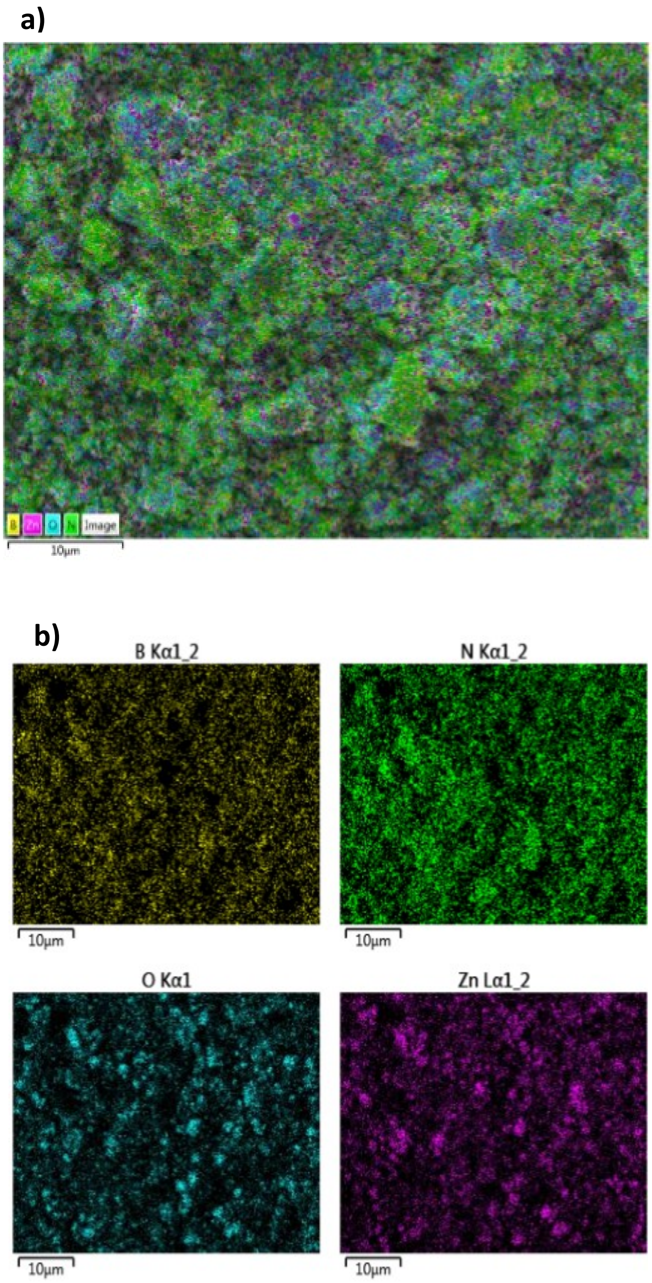

Figure S2. (**a**) EDX image of h-BNNS exfoliated with 1.0 wt % $ZnCl_2$; (**b**) Element mapping images of the h-BNNS exfoliated with 1.0 wt % $ZnCl_2$.
